# Allelic Variants of CRISPR/Cas9 Induced Mutation in an Inositol Trisphosphate 5/6 Kinase Gene Manifest Different Phenotypes in Barley

**DOI:** 10.3390/plants9020195

**Published:** 2020-02-05

**Authors:** Tomas Vlcko, Ludmila Ohnoutkova

**Affiliations:** Laboratory of Growth Regulators, Palacký University & Institute of Experimental Botany, Czech Academy of Sciences, Šlechtitelů 241/27, Olomouc 78371, Czech Republic; tomas.vlcko@upol.cz

**Keywords:** CRISPR, barley mutant, abiotic stress, salinity, phosphate, phytic acid

## Abstract

Inositol trisphosphate 5/6 kinases (ITPK) constitute a small group of enzymes participating in the sequential phosphorylation of inositol phosphate to inositol hexakisphosphate (IP6), which is a major storage form of phosphate in cereal grains. The development of lines with reduced IP6 content could enhance phosphate and mineral bioavailability. Moreover, plant *ITPK*s participate in abiotic stress signaling. To elucidate the role of *HvITPK1* in IP6 synthesis and stress signaling, a barley *itpk1* mutant was created using programmable nuclease Cas9. Homozygous single bp insertion and deletion mutant lines were obtained. The mutants contained altered levels of phosphate in the mature grains, ranging from 65% to 174% of the wild type (WT) content. Homozygous mutant lines were tested for their response to salinity during germination. Interestingly, insertion mutant lines revealed a higher tolerance to salinity stress than deletion mutants. Mature embryos of an insertion mutant *itpk1-2* and deletion mutant *itpk1-33* were cultivated in vitro on MS medium supplemented with NaCl at 50, 100, and 200 mM. While both mutants grew less well than WT on no or low salt concentrations, the *itpk1-2* mutant was affected less than the WT and *itpk33* when grown on the highest NaCl concentration. The expression of all *ITPK*s was induced in roots in response to salt stress. In shoots, the differential effect of high salt on *IPTK* expression in the two *iptk1* mutants was consistent with their different sensitivities to salt stress. The results extend the evidence for the involvement of *ITPK* genes in phosphate storage and abiotic stress signaling.

## 1. Introduction

Barley (*Hordeum vulgare*) a member of the *Poaceae* family was one of the first domesticated crops. Barley belongs among the most important crops globally, being used primarily as animal feed, food, and especially in the brewing industry. One of the drawbacks of the barley-based feeding mixture is poor utilization of phosphate that is present mainly in the organic form of IP6, commonly known as phytic acid (PA). PA is the main storage form of phosphate in cereal grains [1]. Naturally, plant seeds produce phytases, which are a group of phosphatases that hydrolyse PA during germination to make phosphate available for the young seedling. Regarding livestock nutrition, PA is considered an anti-nutrient because of its low digestibility by non-ruminants such as poultry or pigs. Currently, feeding mixtures are supplemented with inorganic phosphate, mostly as calcium phosphate, in order to improve their poor phosphate bioavailability. A more advanced option for supplementing the feeding mixture is the addition of purified microbial phytase that aids PA degradation. Unfortunately, these forms of supplementation are relatively expensive.

There are two pathways of PA synthesis in plants, lipid independent and lipid dependent, which produce PA through sequential phosphorylation of *myo*-inositol or phosphatidylinositol, respectively [2]. The lipid-independent pathway was proposed to be involved mainly in phosphate storage during grain development [3]. On the other hand, the lipid-dependent pathway via phosphatidylinositol intermediates produces molecules active in cell signaling [4]. Disruption of the PA biosynthetic pathway led to decreased production of PA that was accompanied by increased levels of phosphate in the mature seeds of pea [5] or rice [6,7]. Interestingly, an intermediate in PA synthesis, inositol triphosphate (IP3), is an important cell signaling molecule playing a role as a secondary messenger [8,9]. Two approaches have evolved to cope with the issue of poor grain phosphate utilization. The earlier approach involved the development of low-phytic acid (*lpa*) lines using classical mutagenesis [5,10,11,12,13,14,15]. These lines carried mutations in genes encoding enzymes involved in PA biosynthesis, mostly inositol phosphate kinases or *myo*-inositol phosphate synthases. Feeding trials with poultry [16], swine [17,18], sheep [19], and rats [20] confirmed that *lpa* barley lines had better nutritional value, with increased mineral and phosphate digestibility. It was observed in previous studies with several *lpa* mutants that these plants have impaired seed or plant performance [21]. The affected phenotype included reduced germination rate [22] and yield [23] with some seed sterility [24]. These findings indicated that phytic acid and its biosynthetic intermediates are of considerable importance for plant metabolism and development. In the light of the performance of different *lpa* mutants grown at five locations over two seasons, Raboy et al. [25] concluded that out of six known *lpa* traits in barley only a few affected grain yields significantly. Moreover, a moderate reduction of PA content (mutant *lpa2-1*) had no effect on yield. Biotechnological approaches have included the development of transgenic lines over-expressing plant or microbial phytases, usually under seed-specific promoters [26,27,28]. The increased phytase activity in mature grains resulted in improved phosphate level and the phytase over-expressing lines successfully competed with *lpa* mutant lines for increased phosphate digestibility.

*ITPK*s are not only proposed to be involved in the “Pi storage” pathway, but they were also reported to play a role in the response to abiotic stress [29,30]. Drought and salinity represent important abiotic stresses, which can critically influence sustainable yield. Plants perceive abiotic stress via multiple pathways to cope with inhospitable environmental conditions. Plant hormones are essential in signaling during adverse growth and environmental conditions. Drought and salinity stress signaling pathways share a unique signal leading to the accumulation of the plant hormone - abscisic acid (ABA) [31]. Elucidating the crosstalk between stress and hormone signaling pathways is necessary for a better understanding of the processes by which plants respond to abiotic stress. *myo*-Inositol is an important biomolecule in plant metabolism, forming conjugates with plant hormones and acting as a substrate for phosphate accumulation, while isomeric inositols and their O-methyl esters participate in seed desiccation and salt tolerance [32]. Regulation of InsP synthesis by ABA signaling is supported by a report by Aggarwal et al. [33], who showed that ABA controls the expression of PA synthesis genes. There is growing evidence for the involvement of plant ITPKs in abiotic stress signaling. For example, Du et al. [29] reported that a rice *Ositpk2* mutant accumulated significantly less osmolytes, such as proline or soluble sugars, under drought stress conditions than did the wild type. Furthermore, Niu et al. [34] suggested that *OsITL1*, which encodes an ITPK, might be a negative regulator of osmotic stress signaling. Both *OsITPK2* and *OsITL1* are orthologues of barley *HvITPK2*, an intron-containing member of the *ITPK* group. As reported for *GaITPK2* [30], the promoter region of *HvITPK1* contains binding motifs for ABA responsive element and two drought-inducible MYB transcription factors, suggesting its involvement in abiotic stress signaling.

Currently, programmable nucleases have become a widespread biotechnological tool that enables precise genome manipulation. Initially, a transcription activator-like effector was fused with *Fok*I endonuclease to induce breaks in double-stranded DNA [35] and lately, TALE-nuclease (TALEN) fusion was used to create mutations at pre-designed genomic sites of *adh1* in *Arabidopsis thaliana* thus confirming its usability in plants [36]. When a revolutionary clustered regularly interspaced short palindromic repeats (CRISPR)/CRISPR-associated 9 (Cas9) system was introduced to the scientific community [37], it instigated a new epoch of genetic engineering. The Cas9 system was quickly adapted for plant genetic modifications [38]. Jiang et al. [39] and Shan et al. [40] demonstrated that Cas9 can be used to create mutations in dicot as well as monocot plant species. The simplicity of the design along with effectiveness in creating mutations constitute the main assets of the Cas9 system. Unlike TALEN, the Cas9 system can be easily used to target several genomic loci simultaneously by introducing multiple single guide RNAs (sgRNA) thus increasing the mutagenic potential of Cas9. Diverse versions of an expression vector have been proposed from using multiple sgRNA cassettes each with its own U6/U3 promoter [41] to chimeric tRNA/sgRNA constructs, which are processed in the cell [42]. Unfortunately, the Cas9 system possesses an undesirable feature, the so-called off-target activity, by which DNA is cleaved at remote sites similar to the designed protospacer. However, off-targeting can be substantially reduced by the application of paired Cas9-based nickases, which increase specificity and reduce the risk of off-target mutations [43]. Additionally, efforts have been made to increase the specificity and efficacy of Cas9 by altering its amino acid sequence [44,45] or sgRNA sequence [46]. 

Once the desired mutant has been produced by CRISPR/Cas9, it may be appropriate to separate the mutation from the *Cas9* transgene in order to reduce the risk of introducing off-target mutations in subsequent generations or for application in breeding programs, for which transgenic plants are not desirable. In plants, the most economically relevant approaches are simple segregation of the mutation in the progenitor lines or isolation of the desired mutation by crossing. For barley, the old cultivar Golden Promise has been used for the development of most transgenic lines [47], requiring the transmission of the mutation into elite cultivars if practical application was planned. Interestingly, promising research has been published describing the development of transgene-free mutant plants through transient expression without the need for exacting in vitro cultures [48,49], but the efficiency of such methods remains low and optimization of the process is needed. 

In this work, nuclease Cas9 was used to induce mutations in *ITPK1* in barley. Primary transformants were screened for the desired mutation and its heritability was verified in the next generation. The presence of potential off-target sites was also investigated. Furthermore, the impact of the introduced mutation on phosphate content in mature barley grain and its effect on abiotic stress tolerance were examined.

## 2. Results and Discussion

### 2.1. Target Gene Analysis

**Figure 1:**
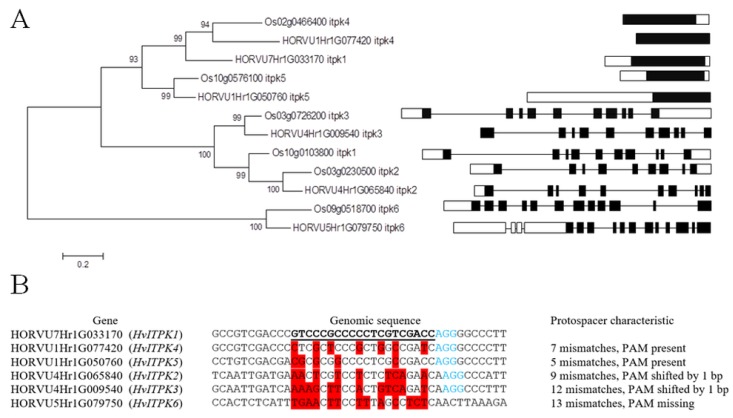
Analysis of barley *ITPK* genes. (**A**) Phylogenetic tree of barley and rice ITPK proteins. The phylogenetic tree was built using amino acid sequences. The scheme of *ITPK* genes illustrates exons (black), introns (joining), and untranslated regions (white). (**B**) Alignment of protospacer sequences within barley *ITPK* genes. Mismatches within the protospacer sequence are highlighted in red, the PAM sequence is shown in blue font.

In the present study, genome-edited barley plants were developed using the CRISPR/Cas9 system [37] adapted and optimized for use in monocot plants [41] in order to prepare barley *lpa* mutants. Not all the genes of the PA synthetic pathway can be considered as ideal targets for silencing. Several mutants in the PA synthesis pathway were described to exhibit weaker phenotype than WT line. For instance, mutants in the genes of an early phase of PA synthesis such as the *myo*-inositol phosphate synthase gene (*MIPS*) exhibited a reduced germination rate [15]. This is consistent with MIPS, which is critical for inositol metabolism [50], having a crucial role in several physiological processes in plant growth and seed development. On the other side, mutation of genes from the late phase of PA synthesis might also have deleterious effects on plant growth, as exemplified by an *A. thaliana* null mutant of *IPK*1, which was not viable [24]. Bhati et al. [51] identified four *ITPK* genes in wheat, whose expression peaked at different stages of grain development suggesting their involvement in phosphate storage. Therefore, genes in the middle part of the pathway were regarded as potential targets to knock-out. Barley *ITPK*s were chosen as a group of candidate targets because a maize *ITPK* gene had been already reported to be also responsible for the *lpa* phenotype if mutated [52]. The barley *ITPK* homologues were identified and analyzed *in silico*. There are six *ITPK* genes in the barley genome that can be classified into two groups. There are three intronless genes (HORVU7Hr1G033170, HORVU1Hr1G050760, and HORVU1HrG077420), while the other genes (HORVU4Hr1G065840, HORVU5Hr1G079750, and HORVU4Hr1G009540) consist of ten or twelve exons (Figure 1A). The nomenclature of the barley *ITPK*s was adopted from the rice nomenclature. The majority of barley genes have a corresponding rice ortholog, except one. Phylogenetic analysis revealed that the barley genome contains three intronless genes in comparison to only two intronless genes in rice. Analogously, rice has four intron-containing genes while there are only three in barley. Close homology between *HvITPK4* and *HvITPK1* suggests that duplication of this gene occurred in barley, although this did not occur in rice. In order to closely investigate the role of the barley *HvITPK*1 gene in PA synthesis and abiotic stress signaling, *HvITPK1* was selected as a target gene for knock-out. *HvITPK1* is an intronless gene coding for a protein consisting of 345 amino acids (AA). The protospacer sequence was designed to maximize Cas9 activity [53] and designed at the beginning of the cds. The secondary structure of the sgRNA was verified using Mfold online tool (http://unafold.rna.albany.edu/?q=mfold), (Figure S1). Cas9 nuclease was directed 181-bp downstream of the *IPTK* start codon (Figure S2A). A multiple sequence alignment of *ITPK* coding sequences was made to identify putative off-target sites in homologous *ITPK* genes, showing varying degrees of nucleotide identity, from only 5 mismatches to 13 mismatches within the protospacer sequence (Figure 1B). 

### 2.2. Barley Transformation

**Figure 2:**
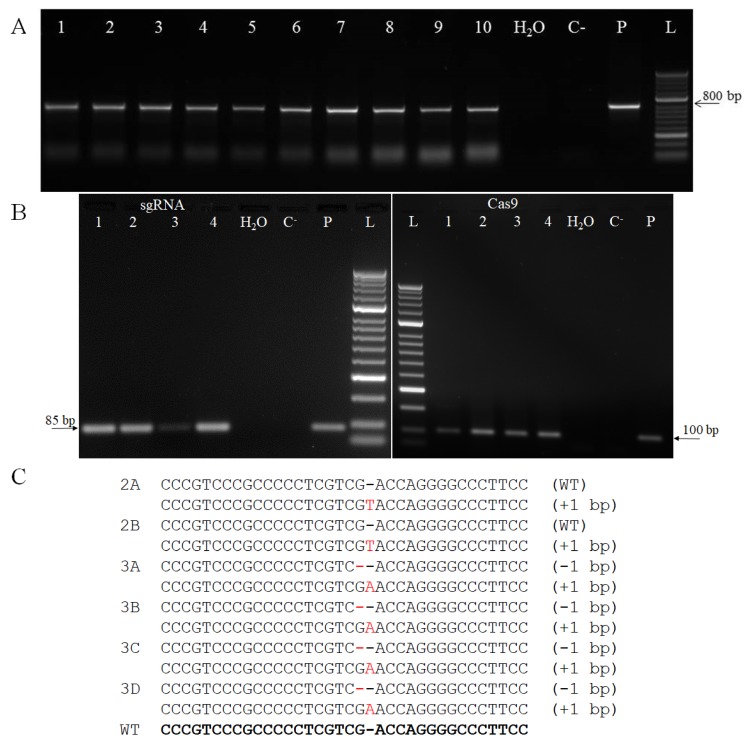
Characterization of primary regenerants at DNA and RNA level. (**A**) Detection of *Cas9* transgene in samples of genomic DNA of T0 plants. L – DNA ladder (HyperLadder 50-bp, Bioline); P – plasmid; C–wild-type plant; 1–10 –samples of T0 plants. (**B**) Detection of sgRNA (left) and Cas9 (right) transcripts in T0 transgenic plants. L – DNA ladder (HyperLadder 50-bp, Bioline); P – plasmid, C^-^ – wild-type plant, 1–4 – cDNA samples of T0 plants, sgRNA transcript product size 85-bp; *Cas9* transcript product size 100-bp. (**C**) Identified mutations in the T0 generation. Labels 2A, 2B, 3A, 3B, 3C, and 3D correspond to independent transgenic events, WT–wild type.

In total, 100 immature barley embryos were used for *Agrobacterium*-mediated transformation with construct pYLCRISPR/Cas9P_ubi_-H. The Golden Promise cultivar, which was used in this study, was selected due to its high capacity to regenerate in tissue culture, which is an essential condition for successful plant transformation [54]. After in vitro cultivation, selection and regeneration, 17 green plants were obtained. Based on the PCR screening of the primary regenerants (Figure 2A), the *Cas9* transgene was detected in 13 plants (independent transgenic events), accounting for transformation efficiency of 13%, which is lower than the average transformation efficiency of 25% reported for this protocol [55]. The expression of the transgenes was verified at the RNA level. Transcripts of both *Cas9* and *protospacer-sgRNA* were detected in the T0 generation of the transgenic plants (Figure 2B).

All 13 T0 transgenic plants were characterized at the genomic level. The target genomic locus amplified by PCR and then Sanger sequenced, indicating genetic modification at the target site in 6 plants, corresponding to 46% editing efficiency. In comparison, a similar efficiency was described by Holme et al. [56], while Kapusi et al. [57] described a higher efficiency at 78%. Lawrenson et al. [58] obtained a lower editing efficiency in the first described barley genome editing experiment, with 23% and 10% in primary transformants. Two types of mutations were identified in the transgenic plants, which contained a 1-bp deletion or insertion (Figure 2C). In 7 T0 transgenic plants, no mutation was detected despite the presence of Cas9 and protospacer-sgRNA transcripts. In the mutant plants, four were bi-allelic, containing an insertion of adenine and a 1-bp deletion concurrently. Additionally, two plants were heterozygous for a substitution at the target site. For monocots, the majority of mutations induced by Cas9 are reported to be single bp indels [41,59,60,61]. It was previously described that nucleotide insertions into the cleavage site are predominantly A or T [41]. In contrast, Sánchez-León et al. [62] reported for wheat that the majority of induced mutations were multiple bp deletions. Moreover, deletions of several hundred bp have also been reported [56], suggesting that the nature of Cas9-induced mutations cannot be simply predicted. Cas9 has also been reported to produce chimeric plants [63]. Therefore, three mutant and two non-mutant T0 plants, which were characterized in the first screening, were additionally selected to evaluate chimerism within primary regenerants. For the analysis, total DNA from five different leaves from each of five selected plants was extracted and the target locus sequenced (Supplementary File S1). No mutation was detected in any of the non-mutant T0 plants. On the other hand, the same bi-allelic mutant constitution was confirmed in additional sequencing of five leaves from plants 3B and 3C, so that these two lines could be classified as uniform mutants. However, plant 2A, which was characterized as a mutant in the initial screening, was not confirmed after additional sequencing, suggesting that it was a chimera. It can be assumed that the mutations detected in plants 3B and 3C occurred early in the callus development. Hence, the biallelic mutant plant labelled 3B was selected for the evaluation of the transmission of the mutation into the next generation. Bi-allelic mutants 3B, 3C, and 3D showed the same mutations in the target locus, 1-bp deletion and insertion, which both caused a shift in the open reading frame. Deletion of G resulted in an occurrence of a premature stop codon in mRNA leading to truncated protein product (139-AA), meanwhile, insertion of A resulted in a prolonged transcript coding for a protein consisting of 368-AA. 

In total, 34 T1 plants, which were the progeny of the T0 3B plant, were analyzed for the genetic constitution of the target locus. In the T1 generation, three genetic variants were observed: bi-allelic mutants and deletion or insertion homozygous mutants (Figure S2B). The bi-allelic mutants accounted for 21 lines, whereas the homozygous 1-bp insertion was present in five plants and the deletion in four lines. Possible off-target sites among barley *ITPK* homologues of *HvITPK1* were investigated. Since *HvITPK3* had more than half mismatching nucleotides within the protospacer and a 1-bp shifted PAM and *HvITPK6*, besides containing substantial mismatches lacked PAM completely, these two genes were excluded from the evaluation of off-target sites. No putative off-target mutation at the predicted sites within other *ITPK* genes was identified in the primary regenerants, as well as in the T1 and T2 generations of plant 3B. Interestingly, transgene-free homozygous mutant barley plants were detected in the T2 generation. Hence, the progeny of plant 3B was used in subsequent experiments. 

### 2.3. Phosphate Analysis

**Figure 3:**
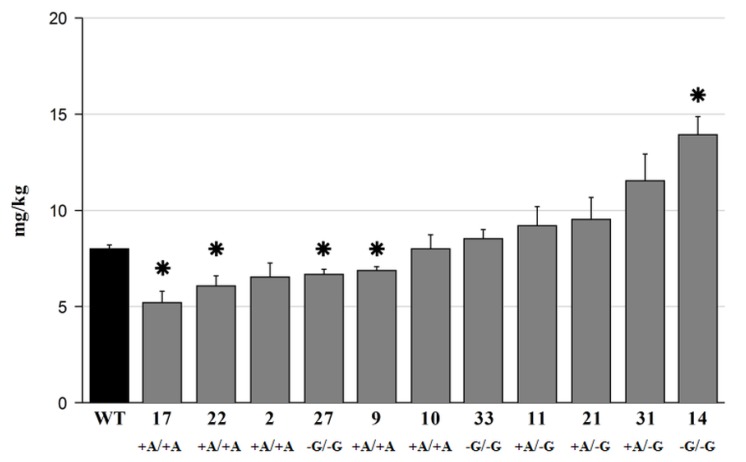
Mature grain phosphate content in *itpk1* mutants. Black column – wild-type plant, grey columns–T2 generation *itpk1* mutant lines. Allelic constitution at the target site for each of the selected parental T1 3B mutant lines shown below. Data represent means (n = 3 ± SD). The asterisks represent significant differences in the phosphate content at *p* < 0.05 compared to WT based on the Aspin-Welsch unequal-variance t-test.

A phylogenetic analysis of rice and barley *ITPK* genes revealed considerable similarity between the two groups of genes. In a recent study, it was reported that a loss of function in *OsITPK6*, a gene from the PA synthetic pathway, could result in a reduction of PA content in mature grains [7]. Hence, it is tempting to presume that the other *ITPK* members can be targeted for knock-outs to alter PA content. A substantial reduction of phytate level in mature grains could potentially be achieved via manipulation of its synthetic pathway without a detrimental effect on plant performance. As it was shown that a reduction of phytate resulted in a proportional increase in inorganic phosphate levels in grains [64,65], the phosphate content was measured in mature grain of the iptk1 mutants. This analysis revealed that the mutations in *HvITPK1* had a diverse effect (Figure 3) and, unexpectedly, did not result in an overall increase of phosphate content in all tested lines. The highest increase in phosphate content, of 74%, was detected in the homozygous deletion mutant *itpk1-14*. In comparison to WT, a majority of the analyzed samples showed only similar or even lower levels of phosphate. Remarkably, a comparison of a biallelic mutant with two homozygous mutant variants showed that these groups generally differed in phosphate content. The homozygous insertion mutants generally contained lower phosphate content in comparison to WT, this decrease reaching up to 35% in line *itpk1-17*. The biallelic mutant lines contained comparable or slightly higher phosphate levels than WT, while the homozygous deletion mutation affected phosphate content the most diversely. Within three tested lines, line *itpk1-33* showed no difference in the phosphate content, while the remaining two lines differed significantly: a decrease by 17% was observed in line *itpk1-27* and a substantial increase by 74% in line *itpk1-14*. Inconsistency in phosphate levels among the studied mutant lines, especially the homozygous lines, could result from incomplete penetrance of the phenotype if the truncated or extended proteins produced from the mutated transcripts retain residual activity. The incomplete penetrance effect could be also associated with a relative position of a mutation within an open reading frame. This was reported recently for chemically (EMS)- and CRISPR-induced barley mutants, which demonstrated more severe phenotype if a mutation was located closer to N terminus [60].

Additionally, there is evidence confirming that mutation in a gene from the PA synthesis pathway affects the expression of the other PA synthesis members. For instance, Kim and Tai [66] showed that *A. thaliana lpa* mutants contained significant changes in gene expression of PA synthetic kinases during seed development. In comparison, Nagy et al. [67] emphasized that mutation in *Atabcc5*, which is a vacuolar PA transporter, resulted in almost doubled expression of some *ITPK* genes, while knock out of a *Phaseolus vulgaris* ABC transporter of PA resulted in a relative decrease in expression of *ITPK* genes [68]. ITPKs can differ substantially in their enzyme kinetic parameters and could phosphorylate both InsP3 and InsP4 [69]. Differential expression profile of wheat *ITPK*s during the grain development [51] suggests that ITPK enzymes are being developmentally -regulated during grain maturation. Considering the different kinetic parameters of ITPK enzymes with their differential expression profile during the grain development, it is tempting to suggest that differential expression of *ITPK* genes is part of a regulatory mechanism to control PA synthesis. The soya ortholog of *HvITPK1* was characterized as a key gene directing the flux of the inositol phosphate (InsP) pool to PA biosynthesis [70] and *TaITPK4*, which is characterized by an increased level of expression at the beginning of seed development [51], shows a high level of similarity with barley *HvITPK1*. It could be presumed that *HvITPK1* might have a similar function in barley by enhancing PA synthesis during early grain development. Hence, the varying level of phosphate among the homozygous mutant and bi-allelic lines, could be explained by impaired PA synthesis potentially coupled with residual protein activity of the HvITPK1 mutant enzymes. 

### 2.4. Abiotic Stress Assay

**Figure 4:**
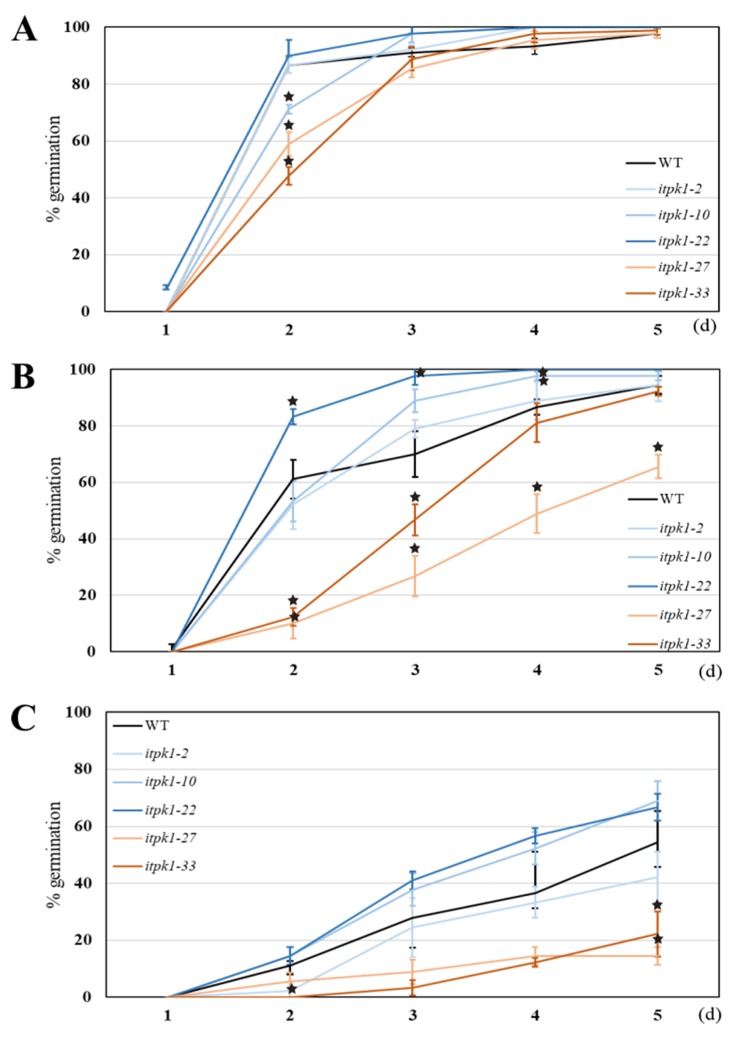
The effect of NaCl on germination of barley *itpk1* mutants. (**A**) Control conditions without NaCl. (**B**) Grains germinated on 100 mM NaCl. (**C**) Grains germinated on 200 mM NaCl. Results for homozygous insertion mutants are displayed with differently shaded blue lines, deletion mutants with orange lines and the WT with a black line. Data represent means (n = 3 ± SD). The asterisks represent significant differences in germination percentage at *p* < 0.05 compared to WT based on the Aspin-Welsch unequal-variance t-test.

In view of above-mentioned evidence for a role for ITPKs in abiotic stress responses, *itpk1* mutants were tested for their phenotypic performance under salinity stress in vivo and *in vitro*. Grains of five homozygous *itpk1* mutants, three insertion and two deletion lines, were germinated on moist filter paper in a growth chamber at 16/8 day/night regime. Under control conditions without salt, all tested lines including WT reached a germination rate of over 90% by the fourth day (Figure 4A). At two days, the two deletion mutants *itpk1-27* and *itpk1-33* and one insertion mutant *itpk1-10* showed a significantly slower germination rate than WT. However, by day three there was little difference between the lines with over 80% of the deletion mutant grain having germinated. Differences in germination rate between the mutant lines were more pronounced on treatment with 100 mM NaCl (Figure 4B). The insertion mutants exhibited similar or even more rapid germination than under control conditions, with *itpk1-22* reaching 100% germination on day 4. On day 3, the three insertion mutants had a higher germination percentage than the WT. In contrast, the germination of the two deletion mutants (*iptk1-33* and *iptk1-27*) was significantly delayed compared with the WT and insertion lines and whereas 90% of *iptk1-33* grain had germinated by day 5, almost 40% of *iptk1-27* grain had still not germinated by this time. The most visible differences in germination rate between the two clusters of homozygous mutant lines were observed after treatment with 200 mM NaCl (Figure 4C). Germination of all lines was notably slower. Two insertion lines, *iptk1-22* and *iptk1-10*, achieved a higher germination rate than the WT, whereas germination of the deletion mutants was significantly less efficient than that of the WT. Application of 300 mM NaCl resulted in total inhibition of germination in WT and *itpk1* mutants (data not shown). 

**Figure 5:**
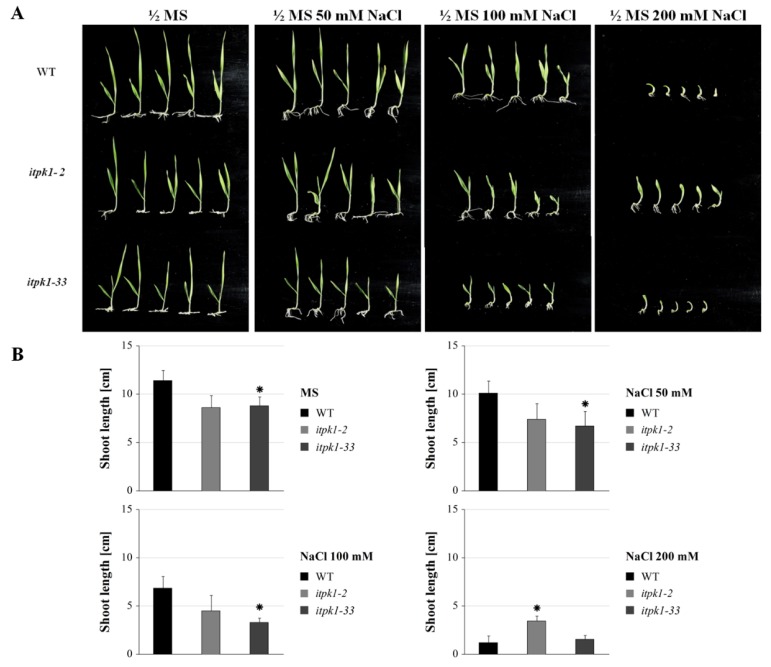
The effect of NaCl on in vitro cultivated barley *itpk1* mutants. (**A**) Barley seedlings from *itpk1-2*, *itpk1-33* and WT groups under control and increasing salt concentration conditions. (**B**) Shoot length after 10-day in vitro cultivation at control and salt stress conditions. The values are the means ± SD (n = 5). The asterisks represent significant differences in the shoot length at *p* < 0.005 compared to WT based on the t-test.

The effect of salt stress on shoot growth for the *iptk1* mutants was evaluated by growing excised mature embryos on cultivation medium in vitro (Figure 5). In order to evaluate the phenotype of two homozygous mutant genotypes, the insertion mutant (+A) *itpk1-2* and deletion mutant (-G) *itpk1-33*, for which there was sufficient grain, were selected. As shown above in Figure 3, the phosphate content in mature grain of these lines did not differ significantly from that of WT. On MS medium without salt, there were differences in shoot growth between WT and the mutants. At the lower salt concentrations, 50 and 100 mM NaCl, WT plants also showed better growth than both mutants, out of which *itpk1-33* seemed to be more affected by salt stress than was *itpk1-2*. This was in contrast to the highest salinity stress conditions (200 mM), at which the growth of all lines was severely inhibited, but *itpk1-2* showed significantly better growth than the WT and *iptk1-33*. Generally, the barley cultivar Golden Promise exhibits tolerance to salinity stress [71]. Thus, although mutant plants exhibited slower growth at 50 and 100 mM NaCl compared to WT, *itpk1-2* showed significantly better growth at the highest NaCl concentration, which supports a potential role for barley *HvITPK1* in salinity stress signaling. Involvement of an intronless member of the *ITPK* group in stress signaling is consistent with the report by Marathe et al. [30], who performed transcription profiling and heterologous expression of the soya ortholog of *HvITPK1* in *E. coli* and showed that this gene is important for response to dehydration and salinity by acting as a stress regulator. In contrast, germination of all *A. thaliana lpa* mutants, including *iptk1*, was more sensitive to salt, osmotic, and oxidative stress than the wild type [66]. In the current study, comparison of both assays showed that there were clear phenotypic differences between mutant lines cultivated under salt stress. These differences were conditioned by the type of induced mutation. Shoot growth was impaired relative to WT in both allelic mutant variants under control and lower salt concentration conditions, while at the highest salt concentration the homozygous insertion mutant *itpk1-2* out-performed WT and *itpk1-33* consistent with its enhanced salt tolerance. This observation was further supported by the germination assay, where the three homozygous insertion lines (*itpk1-2*, *itpk1-10*, and *itpk1-22*) showed higher germination rate than two deletion lines (*itpk1-27* and *itpk1-33*).

### 2.5. Transcription Profiling of ITPK Genes under Salinity Stress

**Figure 6:**
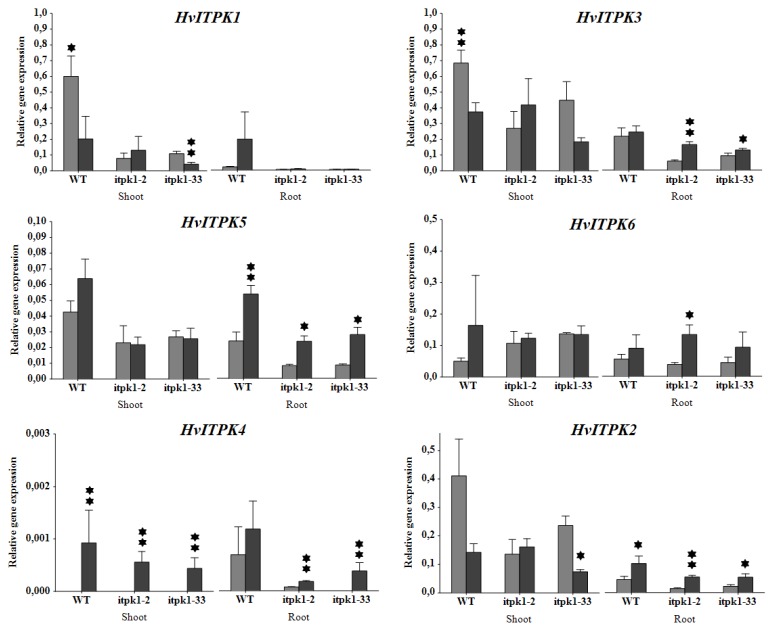
Relative transcript abundance of *ITPK* genes. Light grey – control condition (MS medium), black – salinity stress (200 mM NaCl). The values are means ± SD (n = 3). The asterisks denote significant differences in gene expression between control and stress conditions, for * at *p* < 0.05 and ** *p* < 0.01 based on the Aspin-Welsch unequal-variance t-test.

An important role for inositol phosphate kinases during abiotic stress signaling is supported by the regulation of their expression by ABA [33], a potent abiotic stress signaling hormone. It is possible that enzymes from the initial phase of InsP synthesis might also participate in responses to abiotic stress. MIPS catalyses the initial step of PA synthesis, the conversion of glucose-6-phosphate to *myo*-inositol-3-phosphate. Kaur et al. [72] showed that heterologous expression of *CaMIPS2*, which is an early dehydration-responsive gene in *Cicer arietinum*, in *A. thaliana* led to higher tolerance to salinity and dehydration. In chickpea, the promoter region of *CaMIPS2* contains the CRT/DRE *cis*-regulatory element, which was predicted to play a key role in the regulation of exogenous ABA- or stress-induced expression [72]. Remarkably, the promoter region of *HvITPK1* contains ten copies of this element suggesting that expression of this gene is likely to be under regulation by ABA. Previously, Du et al. [29] demonstrated that knock-out or over-expression of *OsITPK2 (HvITPK2* ortholog) can affect the expression of some of the homologous genes. In the present study, transcript profiling performed on WT and the two *itpk1* (*itpk1-2* and *itpk1-33*) mutants grown on half MS medium with or without supplementation with 200 mM NaCl revealed an altered expression pattern of *ITPK* genes in the mutants in comparison to WT (Figure 6). Under control conditions, expression of *HvITPK1* was higher in shoots than in roots of WT. However, the expression of *HvITPK1* increased in roots, but dropped significantly in shoots under salt stress in WT, suggesting that *HvITPK1* plays a role in abiotic stress signaling. There was weak expression of *HvITPK1* in the leaves of both mutants relative to WT. Moreover, for the *itpk1-33* mutant, but not for *iptk1-2*, a drop in *HvITPK1* expression under salt was observed in shoots as for WT. In contrast, no significant increase in *HvITPK1* expression was noticed in roots of both mutants on salt treatment. It was reported that mutations in a gene open reading frame can result in the suppression of its transcription and translation, although, the residual mRNA level is not predictive of residual protein level [73,74]. 

Gene expression analysis of other *ITPK* genes in roots of WT and both mutants showed that expression of all *ITPK*s was generally increased in response to salt stress. Particularly, the expression of *HvITPK5* and *HvITPK2* was significantly increased in the roots of the mutants and WT. The expression of *HvITPK3* only was not altered in roots of WT in response to salinity, while there was a significant increase in both mutants. In contrast, the expression in shoots of WT and mutants varied. Expression of the remaining two intronless genes, *HvITPK5* and *HvITPK4*, was lower than that of the intron-containing genes. Actually, *HvITPK4,* which is the closest homolog of *HvITPK1*, showed very low or no expression in roots and shoots under control conditions and its expression was induced in the response to salt stress. In shoots of WT, there was an increase in the expression of *HvITPK5* under salt treatment, but this did not occur in the mutants. Previously, *OsITPK2* was characterized as a critical regulator of inositol phosphate-mediated signaling, which was induced by drought, salinity, or abscisic acid [29]. In the present work, a substantial downregulation of its barley ortholog by salinity was noticed in shoots of WT and also in *itpk1-33*. In shoots of WT, there was also a significant decrease in the relative expression level of *HvITPK3* in response to salt. A similar decrease in *HvITPK3* expression was observed in the *itpk1-33* mutant, although this was not statistically significant. In comparison, a decrease in expression of *HvITPK3* and *HvITPK2* after salt treatment was not evident in *itpk1-2*, where the expression increased slightly or was unchanged for *HvITPK3* and *HvITPK2*, respectively. Notably, the differential expression profiles of *HvITPK2* and *HvITPK3* in shoots in response to salt between the two *itpk1* mutants correlate with their different salt sensitivities. The data obtained in this study suggested that i) maintenance of *HvITPK1* expression in shoots of *itpk1-2* mutant might be related to the altered function of its protein product, possibly resulting in higher tolerance of young seedlings to salinity and ii) disruption of one *ITPK* gene can affect the expression of the other members of the *ITPK* group, at least in shoots. 

Taken together, factors such as residual enzyme activity, diverse rate of expression of mutated genes, and varying levels of compensation by altered expression of paralogs, could have an effect on the total phosphate content in the mature barley grains in *itpk1* barley mutants. In a recent study, it was pointed out that different mutations in a gene open reading frame can result in the different phenotype and influence gene function [74]. Due to the diverse effect of the two mutations on the phenotypic manifestation in the studied lines, it was not possible to determine the exact role of *HvITPK1* in abiotic stress signaling. Nevertheless, the observations support the thesis that both homozygous allelic variants have a negative effect on shoot growth under control and weak salinity stress (50 and 100 mM NaCl) conditions. However, at 200 mM NaCl, insertion mutation most likely conditioned salt tolerance in *itpk1-2*. The presence of multiple regulatory and binding elements in the promoter region of *HvITPK1* implies that it may have an important function during abiotic stress signaling. Hence, the potential involvement of barley *ITPK* genes during early responses to drought and salinity stress warrants further investigation. Two allelic variations were studied in this study. Phenotypic differences conditioned by the two alleles suggested that the mutation in *HvITPK1* could affect protein functionality, and in the case of the insertion mutation, this altered function may have enhanced salt tolerance. 

## 3. Materials and Methods 

### 3.1. Design and Cloning of Protospacer

Barley inositol trisphosphate 5/6-kinase (HORVU7Hr1G033170, here referred to as *HvITPK1*) was chosen as a target gene. The protein-coding sequence (cds) of *ZmIPK* [52] was used to blast the barley genomic DNA database (EnsemblPlants) to identify homologous barley *ITPK* genes and rice orthologs. MEGA 6.06 software was used for multiple sequence alignment of all barley *ITPK* cds. Neighbour-joining phylogenetic tree was constructed with protein sequences of barley and rice ITPKs using Clustal W and MEGA 6.06 with 1000 bootstrap replicates. Prior to protospacer design, the target locus in the donor plant material, barley cultivar Golden Promise, was PCR-amplified and Sanger sequenced, on the basis of which the protospacer sequence was designed. The protospacer was sub-cloned into pYLsgRNA-OsU6 and then the cassette containing sgRNA under the small U6 promoter was cloned into the expression vector pYLCRISPR/Cas9Pubi-H according to the protocol by Ma et al. [41]. Correct integration of the sgRNA cassette was verified by digestion with *Mlu*I, and by Sanger sequencing, (Figure S3). The vector harbouring sgRNA with protospacer sequence was transformed into *Agrobacterium tumefaciens* strain AGL1 by electroporation. Standard bacterial inoculums with OD_600_ of 1.0 were prepared and stored at −80 °C. 

### 3.2. Barley Transformation

The barley model cultivar Golden Promise was used for transformation according to the protocol by Harwood [55]. Barley plants were grown in the greenhouse with a 12-h photoperiod and 15 °C for 10–12 weeks. For the identification of transgenic plants, genomic DNA from young leaves was extracted according to Edwards et al. [75] and analyzed for the presence of Cas9 transgene by polymerase chain reaction (PCR). The genotyping primers are listed in Table S1. For the PCR reaction, a premix REDTaq^®^ ReadyMixTM PCR Reaction Mix (Sigma-Aldrich, St. Louis, MO, USA) was used. The reaction was started with 5 min denaturation at 95 °C, followed by 40 cycles of 30 s denaturation, 30 s annealing at 58 °C and 1 min elongation at 72 °C. The PCR products were separated on a 1% agarose gel stained with ethidium bromide. 

### 3.3. Detection of the Cas9 and sgRNA Transcripts

Total RNA was extracted from young leaf tissue of T0 plants using a Total RNAqueous Kit (Thermo Fisher, Vilnius, Lithuania) and treated with Turbo DNase according to the manufacturer’s protocol. The concentration of RNA was assessed spectrophotometrically (DeNovix Spectrophotometer DX-1), 1 μg of RNA was reverse transcribed using poly-T primers and RevertAid H minus Reverse Transcriptase (ThermoFisher). The cDNA was diluted ten times with nuclease-free water and amplified with specific primers for Cas9 and sgRNA transcripts, listed in Table S1. In the case of sgRNA, the forward primer was derived from the protospacer sequence and the reverse primer was located in the guide RNA sequence. PCR conditions were the same as used for genotyping the T0 plants, except for an annealing temperature of 60 °C for 20 s and a shorter extension time of 30 s. 

### 3.4. Genotyping of Primary Regenerants and their Progeny

Plant material was collected two weeks after the transfer of regenerated plants into pots. Genomic DNA was extracted from young leaves of the transgenic plants as described [75] with two biological replicates, two different leaves, per plant. To evaluate the induction of target and off-target mutations, primer pairs for amplification of target and off-target loci in the closest homologues of *HvITPK1* within the group of *ITPK* kinases were designed, verified and used for genotyping. All the primers used for genotyping of target and off-target loci are listed in Table S1. The target and off-target loci in T0 transgenic plants were PCR-amplified and the PCR products were Sanger sequenced. For decoding and evaluation of the sequencing chromatograms, DSDecode online tool [76] was used. For the analysis of heritability of the target mutations and induction of off-target mutations in the next generation, the progeny of the bi-allelic mutant line 3B was genotyped in the T1 and T2 generations. 

### 3.5. Seed Phosphate Analysis

For phosphate analysis in mature grain, progenitor lines originating from the bi-allelic mutant plant T0 3B were selected. Mature barley grains (T2 generation) of eleven mutant T1 3B lines were harvested and homogenized to a fine flour. Lines containing a homozygous 1-bp insertion were 17, 22, 2, 9, and 10; lines carrying a homozygous 1-bp deletion were 27, 33, 14. The remaining lines 11, 21, and 31 were progeny of bi-allelic mutants. Phosphate analysis was done according to the method of Vaculova et al. [77]. Briefly, 50 mg of homogenized sample was mixed with 0.5 mL of 0.4 M HCl and incubated overnight at 4 °C. Then, the samples were centrifuged 10 min at 6000 g and the supernatant was used for analysis. Phosphate analysis was based on the colorimetric assessment of the complex formed from the reaction of Chen’s reagent [78] with phosphate. Absorbance was measured at 822 nm after two-hour incubation with Chen‘s reagent and the phosphate concentration was determined from a calibration curve. Technical and biological measurements were made in triplicate. The mean value with standard deviation was plotted on a graph.

### 3.6. Abiotic Stress Experiments

#### 3.6.1. Seedling Growth Assay

The homozygous T2 mutant lines, deletion mutant *itpk1-2* and insertion mutant *itpk1-33* were selected for in vitro testing of their response to salinity stress. Mature grains of WT as a control and *itpk1* mutants were soaked in sterile water overnight, the embryos dissected and collected in a tube with fresh sterile water. The embryos were sterilized for 1 min in 1% sodium hypochlorite solution with agitation and then washed twice in sterile water. Solid half MS medium with 10 g/L sucrose, Phytoagar (Duchefa) 6 g/L, with the pH adjusted to 5.8 was prepared in flasks. The media were supplemented with NaCl at 50, 100, or 200 mM. Pure half MS medium without NaCl supplement was prepared as a control. The sterilized embryos were placed on the surface of the medium, five embryos of each mutant line and WT per treatment. The flasks with embryos were placed in a growth cabinet and cultivated at 24 °C and a day/night regime of 18/6 h. After two weeks, the phenotype of the seedlings was evaluated by measuring the height of the seedlings.

#### 3.6.2. Germination Assay 

Homozygous T2 mutant lines, deletion mutants *itpk1-2*, *itpk1-10*, *itpk1-22*, and insertion mutants *itpk1-33* and *itpk1-27*, were selected for in vivo testing of their response to salinity stress during germination according to the published protocol [79] with a few adjustments. Grains were sown on 0, 100 or 200 mM NaCl at 30 per plate in three replicates per treatment for each mutant line and WT. Grains were placed on moist filter paper in Petri dishes and kept in the dark at 4 °C for three days. Then, the plates were transferred into the growth chamber under a 16/8 h day-night regime and a constant 23 °C. Germination was assessed daily for 5 days. Grains were considered germinated when the primary root reached a length of approximately 0.5 cm. 

### 3.7. Expression Profiling of Barley ITPK Genes 

Total RNA from young seedlings of WT and homozygous mutant lines *itpk1-2* and *itpk1-33* cultivated in vitro on pure half MS medium and medium supplemented with 200 mM NaCl was extracted and 500 ng of total RNA was reverse transcribed as described above. Total RNA was extracted from roots and leaves separately. The cDNA samples were diluted five times with nuclease-free water. Three biological replicates, where each replicate was represented by one plant, were prepared. *ITPK* transcript levels were determined by qRT-PCR using the primers listed in Table S1. The reaction mixture comprised SsoAdvanced^TM^ Universal SYBR^®^ Green Supermix (BioRad, Hercules, CA, USA), half of the reaction volume, 0.4 μL of each 10 μM primer solution, 1 μL of diluted standard or cDNA sample, and water to 10 μL. CFX96 Touch^TM^ Real-Time PCR Detection System (BioRad) was used for the qPCR analysis. Cycling conditions were initial denaturation at 95 °C for 3 min followed by 40 cycles of 10 s denaturation at 95 °C and 30 s of annealing and amplification step at 60 °C. Melting analysis of the PCR products was performed afterwards to assess the specificity of the primers. PCR products were then separated in agarose gel to further confirm the product specificity (Figure S4). The dilution series of standards for each *ITPK* gene and Elongation factor 1-α (*EF1*- α), Actin2 (*ACT*), and Ubiquitin (*UBI*), which were used as the reference genes, were prepared as follows: The standards were PCR-amplified from freshly prepared cDNA from non-transgenic barley, the PCR products were purified using paramagnetic beads (Agencourt^®^ AMPure XP, Beckman Coulter) and subsequently diluted to concentrations ranging from 10^−3^ – 10^−10^-fold purified product. The standards were run in technical duplicate and mean Cq values were used to create the standard curves. The amplification efficiency was calculated from the slope of the standard curve, (Figure S5). The plant samples were run in technical duplicate. For evaluation of gene expression, the ΔCt method was used applying the mean Ct value from three reference genes to normalize gene expression.

### 3.8. Statistical Analysis

Statistical analyses were performed using NCSS 9 software. The experimental data are presented with the standard deviations based on three to five replicates. For the phosphate measurements, each mutant line was compared with WT. The Aspin-Welsch unequal-variance t-test was used to test significant differences (*p* < 0.05) in phosphate content and germination percentage. For shoot length, the Student’s t-test was employed. The values were the means of five replicates. The significant differences in the shoot length compared to WT were set at *p* < 0.005. Differential gene expression was analyzed using the Aspin-Welsch unequal-variance t-test. 

## 4. Conclusions

In summary, barley homozygous mutants for *HvITPK1* gene were developed by CRISPR/Cas9. Analysis of mutant progenies confirmed stable heritability of the mutation as well as expected segregation associated with the development of transgene-free homozygous mutant lines. Phenotypic differences between homozygous insertion and deletion lines were observed; particularly in the phosphate content of mature grains and in reaction to salinity stress during germination. Additionally, the differences were confirmed at the RNA level by transcript profiling of the *ITPK* genes in the mutant lines in response to salt stress. The conducted experiments indicated that *HvITPK1* participate in processes related to PA synthesis and salinity stress response, thus confirming *HvITPK1* as a functional gene.

## Supplementary Materials

The following are available online at https://www.mdpi.com/2223-7747/9/2/195/s1, Figure S1. Secondary structure of Protospacer-sgRNA. Figure S2. Characterization of target genomic sequence in *HvITPK1*. Figure S3. Digestion of expression vector by *Mlu*I. Figure S4. Verification of qRT-PCR primers specificity. Figure S5. Calibration curves for internal control and *HvITPK* genes used in qRT-PCR. Table S1. List of primers used for genotyping and qRT-PCR. File S1. Genotyping of T0 plants.
