# Supplementary material for: Allelic Variants of CRISPR/Cas9 Induced Mutation in an Inositol Trisphosphate 5/6 Kinase Gene Manifest Different Phenotypes in Barley
Source: Plants (Basel). 2020 Feb 5;9(2):195. doi: 10.3390/plants9020195 (PMC7076722; doi:10.3390/plants9020195)
Supplement: Supplementary file 1 [file plants-09-00195-s001.zip › Supplement/Figure S3.docx]

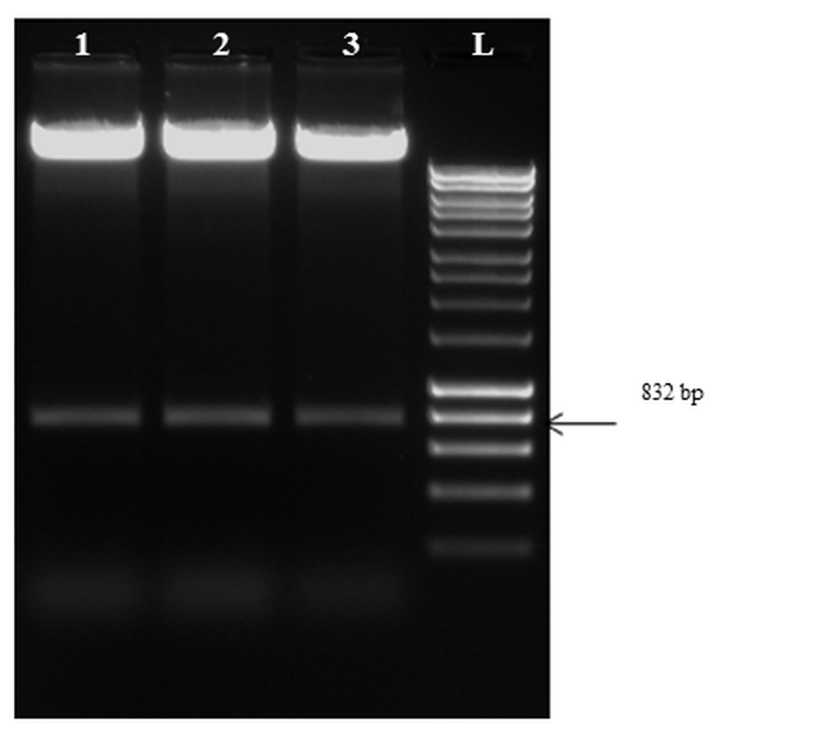


**Figure S3. Diagnostic digestion of the expression vector with sgRNA cassette**

L – DNA ladder (HyperLadder 50 bp, Bioline); 1 – 3 expression vector pYLCRISPR/Cas9Pubi-H harbouring the sgRNA cassette, expected size of cut cassette 832 bp
