# Supplementary material for: Allelic Variants of CRISPR/Cas9 Induced Mutation in an Inositol Trisphosphate 5/6 Kinase Gene Manifest Different Phenotypes in Barley
Source: Plants (Basel). 2020 Feb 5;9(2):195. doi: 10.3390/plants9020195 (PMC7076722; doi:10.3390/plants9020195)
Supplement: Supplementary file 1 [file plants-09-00195-s001.zip › Supplement/Table S1.docx]

| Gene |  | Sequence (5’ – 3’) | Amplicon [bp] |
| --- | --- | --- | --- |
| Cas9 | *Genotyping* | TTCGCTACTGTTCGCAAGGT | 811 |
|  |  | GGTGGATGAGAGTAGCGTCG |  |
|  | *RT-PCR* | CGACGCTACTCTCATCCACC | 100 |
|  |  | CTTTTTGGTGGCAGCAGGAC |  |
| sgRNA | *RT-PCR* | CCCCTCGTCGACCGTTTTAG | 85 |
|  |  | CGACTCGGTGCCACTTTTTC |  |
| HORVU7Hr1G033170 | *Sequencing* | ACGACCTCCTCACACCTACA | 301 |
| *Hvitpk1* |  | GGAGACGACGTCGAGCATG |  |
|  | *qRT-PCR* | TCTTCGGCGACATGATTCGT | 83 |
|  |  | AACGTTGGAGGCTTCGGTAG |  |
| HORVU1Hr1G050760 | *Sequencing* | GCCCAAGAAGCAGAACAGC | 451 |
| *Hvitpk5* |  | GTGGTAGACGAGGGACATCTT |  |
|  | *qRT-PCR* | TGCCTGGCTACGAGATTGTC | 88 |
|  |  | CTCTGCTCCTCCTCCTCCTT |  |
| HORVU1Hr1G077420 | *Sequencing* | TCAGTCGAGCGATCATGGTG | 754 |
| *Hvitpk4* |  | TCCTTGTCCACCAGCCCTG |  |
|  | *qRT-PCR* | TCCAAAGCAGGTGAGAGCAG | 116 |
|  |  | GGCAAACGCTTGGAAGGATC |  |
| HORVU4Hr1G065840 | *Sequencing* | GACCGAGTATTCAGGTGGCC | 529 |
| *Hvitpk2* |  | TTACCTCCAGAATCCGCTGC |  |
|  | *qRT-PCR* | GCTTCCTCCGAGACCACTTC | 110 |
|  |  | ACCGGTCATTAGCTCCAAGC |  |
| HORVU5Hr1G079750 | *qRT-PCR* | TTGAAGACCCTTCCAGTGGC | 101 |
| *Hvitpk6* |  | CGTTTTGCAGCCTCTTCCAC |  |
| HORVU4Hr1G009540 | *qRT-PCR* | ACCTTGCGGATTGTCATGGT | 82 |
| *Hvitpk3* |  | ACCGCAGCTGGTATTGACAA |  |
| HORVU4Hr1G056830 | *qRT-PCR* | GAAGATGATTCCCACCAAGC | 107 |
| *EF1-α* |  | TGACACCAACAGCCACAGTT |  |
| HORVU4Hr1G008310 | *qRT-PCR* | TCGTGAGAAGATGACCCAGA | 122 |
| *Actin2* |  | CCGAGTCCAGCACAATACCT |  |
| HORVU3Hr1G080790 | *qRT-PCR* | GCTTATCATGGCTTCAAAACGTA | 98 |
| *Ubiquitin* |  | ACATATCCTCGCCAGAAGGAC |  |

**Table 1**. List of primers used for genotyping and qRT-PCR.
