# Supplementary figures and images for: Allelic Variants of CRISPR/Cas9 Induced Mutation in an Inositol Trisphosphate 5/6 Kinase Gene Manifest Different Phenotypes in Barley
Source: Plants (Basel). 2020 Feb 5;9(2):195. doi: 10.3390/plants9020195 (PMC7076722; doi:10.3390/plants9020195)

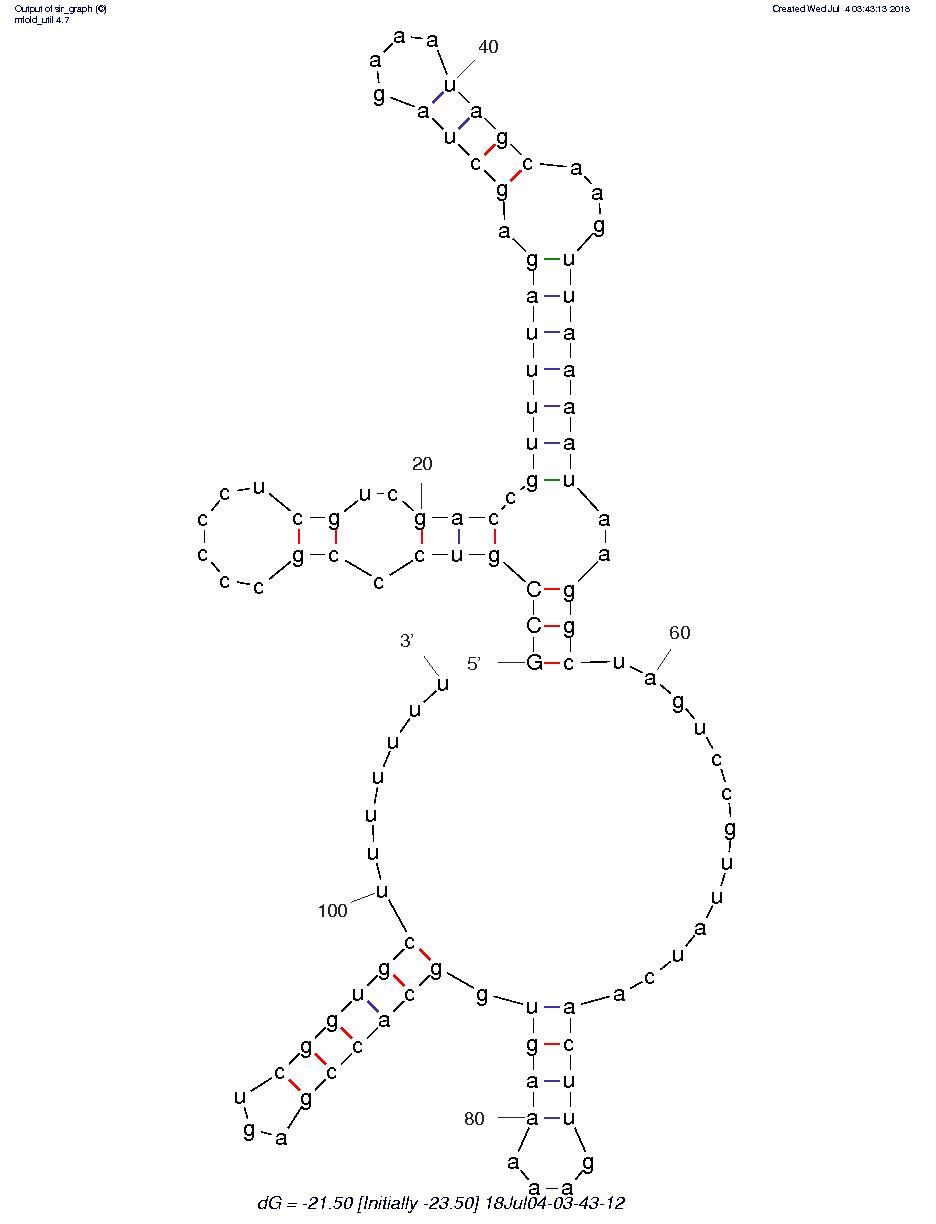

Supplement: Supplementary file 1 [file plants-09-00195-s001.zip › Supplement/Figure S1.jpg]

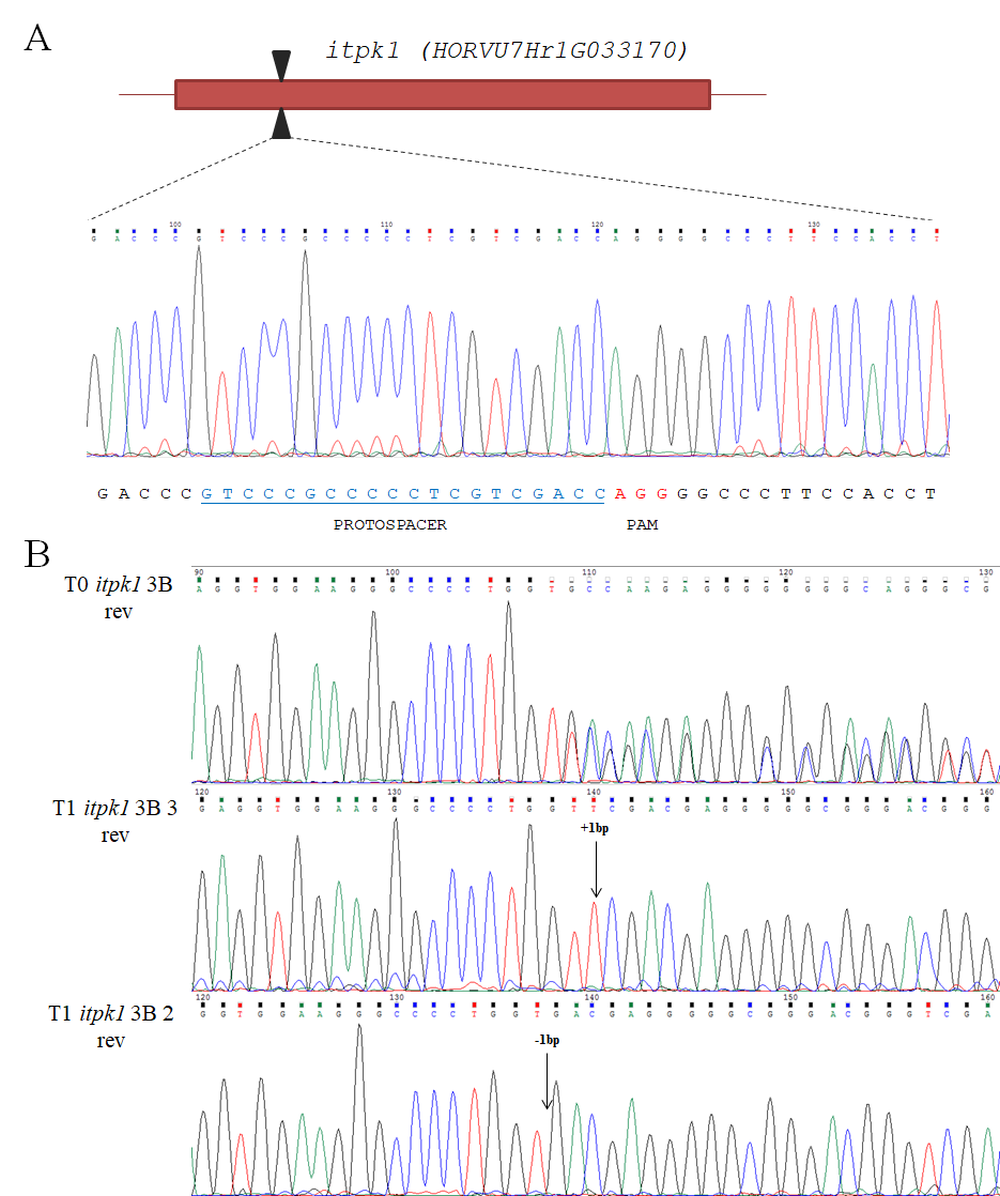

Supplement: Supplementary file 1 [file plants-09-00195-s001.zip › Supplement/Figure S2.tif]

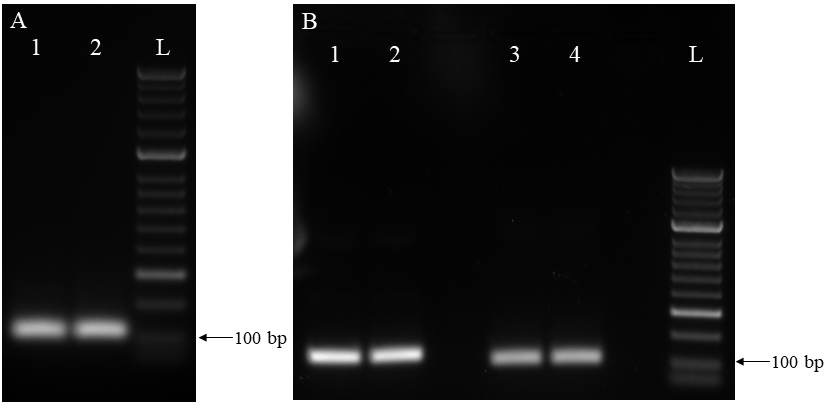

Supplement: Supplementary file 1 [file plants-09-00195-s001.zip › Supplement/Figure S4.docx]
